# Neuritis of the Peripheral Nerves, III

**Published:** 1891-06-06

**Authors:** J. Michell Clarke

**Affiliations:** Assistant Physician Bristol General Hospital


### NEURITIS OF THE PERIPHERAL NERVES.—III.

By J. Michell Clarke, M.A., M.B., M.R.C.P., Assistant Physician Bristol General Hospital.

The following case is an illustration of a rare accident during childbirth, namely, of the paralysis of the external popliteal nerve from pressure on the sciatic nerve within the pelvis. The patient, a woman of 22 years of age, stated that when in labour on November 5th, 1890, she became paralysed in her right leg and thigh. Since then she had gradually improved, recovering power in the knee and ankle, but the foot still remained weak and almost useless. This was her third confinement; she was delivered with the aid of forceps; the two previous children died during birth. There was no history or evidence of syphilis; beyond attacks of dyspepsia, she had had no previous illness of any kind. At first she suffered from very severe shooting pains along the course of the sciatic nerve in the thigh passing down to the leg, and for the first day or two after the confinement the leg seemed quite dead and cold. There was still loss of sensation in the sole of the foot, and some tingling sensations in the toes, but apart from this the pains and the numbness in the thigh and leg had quite ceased. On examination she was anæmic but well nourished. The right foot was dropped, and when it was placed flat on the ground she could not raise it, nor extend the toes in the least degree; abduction of the foot from the middle line was also lost. On the other hand, flexion of the foot, and toes, and the lateral movement inwards of the foot were normal. The walk was characteristic when, the left foot supporting the weight of the body, the right heel was raised. The elevation of the toes and foot, which normally should now take place by the contraction of their proper extensors to allow the foot to swing forwards, did not occur, the foot therefore dropped and the extensors of the knee still further contracted to overcome the difficulty, so that there was thus the "high stepping" gait in one leg only. There was no obvious wasting of the muscles. Electrical examination showed that no reaction could be obtained to the faradic or constant current for the peroneal nerve nor for the muscles supplied by it. The knee-jerks were normal. The plantar reflex absent on the right side, present on the left. Tender points existed at the lower border of the gluteal fold in the mid-line of the thigh, and behind the head of the fibula. There was nearly complete loss of sensation over the contiguous borders of the great and second toes (dorsum) both to touch and pain; slight anæsthesia to touch over the rest of the right great toe (dorsal surface) and over the sole of the foot. Under treatment she has regained some power in the affected muscles, but they are still weak, and the foot still drops, though not quite to the same extent as at first.

